# Self-Healing and Joule Heating of Electrically Conductive CNT/BIIR Composite Films

**DOI:** 10.1021/acsomega.6c01073

**Published:** 2026-05-01

**Authors:** Sang-Yu Park, Hee Jin Kim, Seung Beom Kang, Cheol-Min Yang, Yuree Oh, Young-Kwan Kim, Ji-Young Hwang, Min Wook Lee

**Affiliations:** † Innovative Carbon-Bio-Convergence Lab., 636233 Korea Carbon Industry Promotion Agency (kcarbon), 110-11 Ballyong-ro, Deokjin-gu, Jeonju 54853, Republic of Korea; ‡ Institute of Advanced Composite Materials, 58975 Korea Institute of Science and Technology, Chudong-ro, Bongdong-eub, Jeonbuk 55324, Republic of Korea; § Department of Chemistry, 34942 Dongguk University-Seoul Campus, 30 Pildong-ro, Jung-gu, Seoul 04620, Republic of Korea

## Abstract

This study offers a facile, scalable, and cost-efficient route for the fabrication and repair of a conductive elastomer. Uniform CNT dispersion within the BIIR matrix was obtained through a solution-assisted process by using optimized solvent and dispersant combinations. The resulting CNT/BIIR composites achieved a minimum sheet resistance of 3.33 ± 0.20 Ω/sq and an electrical conductivity of 3.93 ± 0.23 S/cm. Upon electrical stimulation, the composites generated rapid heat through the Joule effect, reaching a maximum temperature of 194 °C, which enabled electrical stimulus-induced self-healing after physical damage. Posthealing, the mechanical and electrical properties recovered to 69 and 354% of their original values, respectively. Furthermore, a solution-spraying repair approach facilitated the effective restoration of electrical pathways and maintained uniform heating performance on curved substrates.

## Graphical abstract

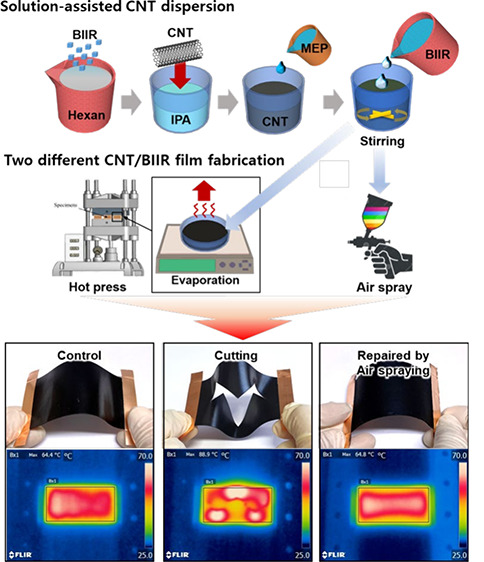

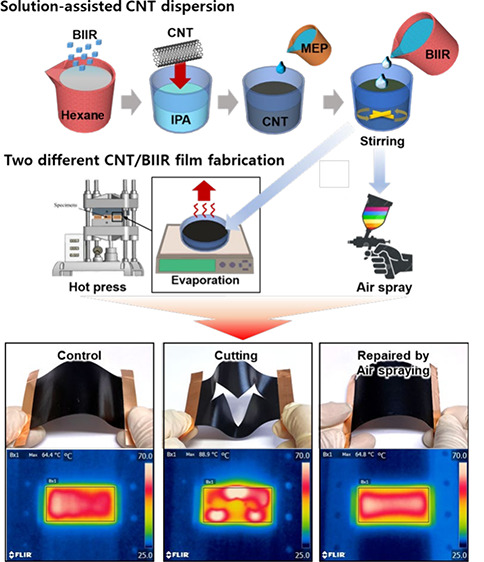

## 1 Introduction

Chemical pollutants, mechanical forces, UV radiation, and various environmental factors can damage products across various industries, such as automotive, aerospace, construction, and electronics. Cracks that begin from minor damage gradually propagate and cause failure, leading to rapid decreases in mechanical, thermal, and electrical properties, and an overall decline in quality.^1–3^ Self-healing materials that can treat cracks or weld themselves have been developed to overcome these problems. With the increasing demand for durable and sustainable products, the development of advanced materials with self-healing capabilities has emerged as a significant issue.^4^

Various materials such as metals,^5^ ceramics,^6^ and concrete materials^7^ have been investigated to present self-healing capabilities, but elastomeric polymers are the most common types of self-healing materials. Nissan’s self-healing paint, “Scratch Shield”,^8^ and Apple’s most recently introduced foldable screen with self-healing properties^9^ are also made of elastic polymers. Among these, rubber-based materials have been widely used in various applications, owing to their flexibility, adaptability, and tunable properties. Recent studies have shown that rubber systems can be engineered for improved mechanical performance,^10^ thermal regulation,^11^ and energy-efficient functionality.^12^ Accordingly, rubber elastomers are promising candidates for self-healing systems because their backbone functional groups provide an intrinsic ability to form reversible bonds and reconstruct the network.^13^ The healing mechanism is generally based on the dynamic bonding-debonding process in supramolecular chemistry. Namely, the cut ends of rubber segments are rearranged by dynamic ionic association and physically cross-linked with each other. Therefore, BIIR enables repeatable self-healing without external agents, unlike capsule-based systems^1^ requiring additional healing agents for repeated healing and swelling^14^/hydrogel-based systems^15^ that depend on external solvent or moisture.

Brominated isobutylene isoprene rubber (BIIR) is a representative example of commercially available synthetic rubber elastomers and self-healing material, which is an isobutylene-isoprene copolymer containing reactive bromine.^16^ It has good physical strength, vibration damping, low gas permeability, and resistance to aging and weathering from environmental exposure.^17^ Its valuable properties make it a widely applied material for several commercial products, such as automobile tires, protective suits, and membranes.^18^ However, a slow self-healing rate and high trigger temperature (about 100 °C) limit the expansion of its application.^19,20^

Therefore, it is an important issue to accelerate the self-healing process of polymeric composites in response to external stimuli like temperature, UV light, electrical stimuli, pH, and humidity for their practical applications as stimuli-responsive self-healing polymers.^21^ There have been many efforts to develop polymeric composites with stimuli-responsive self-healing properties by incorporating functional carbon nanofillers, such as carbon black, graphene, and carbon nanotube (CNT), to enhance their properties and responsiveness to external stimuli.^22^ Especially, CNTs are a promising candidate for functional filler for stimuli-responsive self-healing polymeric composites due to their superior electrical conductivity compared to copper, higher thermal conductivity than diamonds, and mechanical properties thousands of times greater than steel.^23^ In addition, their electrical conductivity can induce heating through the Joule heating effect, making them applicable as an efficient stimuli-responsive filler for self-healing polymeric composites with electrical stimuli.^20,24^ Despite those strong potentials, the inadequate dispersion and heterogeneous distribution of CNTs in the polymer matrix have limited their widespread applications in stimuli-responsive self-healing polymer composites.^25,26^

In this study, we fabricated BIIR-based self-healing conductive elastomer composites using highly dispersible CNTs in the BIIR matrix to provide excellent electrical conductivity, mechanical properties, and stimuli-responsive heat generation. The resulting CNT/BIIR composite exhibited a superior thermal conductivity of 0.217 W/m·K, a tensile strength of 2.32 ± 0.23 MPa, an electrical conductivity of 3.93 ± 0.23 S/cm, and a Joule heating rate of 102 ± 21.9 °C/min compared to those of the BIIR matrix (0.041 W/m·K, 0.19 ± 0.04 MPa, and insulating properties). By applying electrical stimulus to the CNT/BIIR composites, their temperature increased rapidly and led to efficient electrical stimuli-responsive self-healing behavior from physical damage, restoring their mechanical and electrical properties to 0.64 ± 0.08 MPa and 2.23 ± 0.42 S/cm, which are 69% and 354% of their original properties. These results indicated that the CNT/BIIR composites provide excellent physicochemical properties as well as electrical stimuli-responsive self-healing characteristics. The process could facilitate the healing of the film in a damaged area by applying a spraying method using a simple and inexpensive solution. It is possible that our self-healing process, with its easy use and excellent economic feasibility, could be applied to various industrial fields.

## 2 Materials and Methods

### 2.1 Materials

Thin-walled carbon nanotubes (CNTs >98.5%, 6–9 nm diameter, 100–200 μm length) were obtained from JEIO Co., Ltd. (Incheon, Republic of Korea). BIIR and silicone fluid (MEP) were provided by Lanxess (Cologne, Germany) and KCC (Bongdong, Republic of Korea), respectively. All chemicals, including isopropyl alcohol (IPA), hexane, and other organic solvents, were purchased in high-performance liquid chromatography (HPLC) grades with >99.9% purities from Sigma-Aldrich (St. Louis, MO, USA).

### 2.2 Fabrication of CNT/BIIR Composites

The fabrication process of the CNT/BIIR composite is illustrated in Figure 1. To obtain a homogeneous mixture, 4 w/v% of BIIR in 100 mL of hexane was first prepared, stirring at 80 °C overnight in a tightly sealed vessel to minimize solvent evaporation. Each CNT solution with 0, 0.25, 0.50, 0.75, and 1.00 w/v% of CNTs in 100 mL of IPA was prepared after ultrasonication for 30 min. Then, MEP was added to a final concentration of 4 w/w% and ultrasonicated for 10 min. The prepared BIIR solution was slowly added to the well-dispersed CNT solution, and then 100 mL of hexane was added to the mixture, followed by vigorous stirring for 30 min. The compositions and corresponding sample names of the final CNT/BIIR composites are summarized in Table 1. Used solvents, IPA and hexane, were evaporated under reduced pressure. Solid CNT/BIIR composites were subjected to the custom-built hot press at 120 °C with 4000 psi for 10 min, using a 0.5mm spacer (Hando, Offenbach, Germany).

**Table 1:** Sample Names and Corresponding Compositions of the CNT/BIIR Composites

**sample name**	**CNT (g)**	**BIIR (g)**	**MEP (g)**	**CNT/BIIR (wt %)**
**CNT0**	0	4	3.28	0.00
**CNT0.25**	0.25	4	3.29	5.88
**CNT0.50**	0.50	4	3.30	11.10
**CNT0.75**	0.75	4	3.31	15.79
**CNT1.00**	1.00	4	3.32	20.00

**Figure 1:**
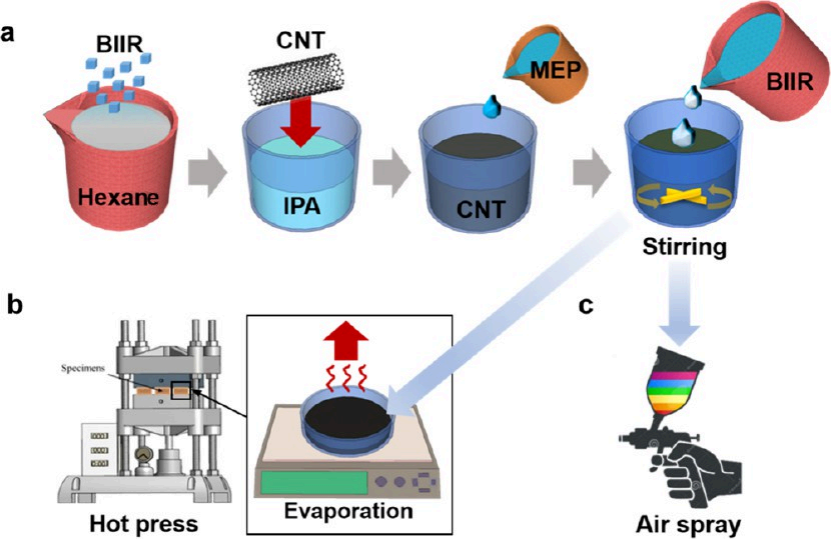
Schematic illustration. (a) Fabrication of a CNT/BIIR solution. Fabrication of CNT/BIIR composite by (b) hot pressing and (c) air-spraying.

### 2.3 Fabrication of the Composite Film by the Air-Spray Method

First, 0.25 and 0.50 w/v% of CNTs with 0.2 v/v% of MEP were homogeneously dispersed in 100 mL of hexane by ultrasonication. Then, the separately prepared 4% w/v BIIR solution in 100 mL of hexane was slowly added to the CNT solution and mixed by vigorous stirring for 30 min. The resulting spray solutions were named S-CNT0.25 and S-CNT0.50. The final mixed solution was sprayed using a commercial air gun (P827, Bluebird, China) onto polyimide film (120 × 50 mm), with a standoff distance of 50 mm from a spray nozzle under a pressure of 60 psi at room temperature.

### 2.4 Characterization

A digital camera (800D, Canon, Japan) was used to take photos of the solutions’ precipitation and stable dispersion. The morphology of each prepared sample was observed using field-emission scanning electron microscopy (FE-SEM; Sigma 500, Zeiss, Germany) at 3 kV. For SEM analysis, the samples were sputter-coated with 10 nm of platinum before analysis to avoid charging effects and better microstructures. The cracked or pore structure of prepared composites was visualized using a nondestructive 3D image obtained by X-ray Microscopy (XRM; Xradia 520 Versa, Zeiss, Germany). The images were acquired with an isotropic voxel size of approximately 0.05 μm.

Thermal properties were evaluated using thermal gravimetric analysis (TGA; Q50, TA Instrument, US), and derivative thermogravimetric analysis (DTG) under a N_2_ atmosphere at a flow rate of 90 mL/min from 40 to 930 °C with a rate of heating of 10 °C/min. Differential scanning calorimetry (DSC; Q20, TA Instrument, US) was used under a N_2_ gas flow rate of 50 mL/min at a heating temperature of −60 to 250 °C at the rate of 10 °C/min. The thermal diffusivity was measured using Laser Flash Analysis (LFA; 467 HT hyperflash, NETZSCH, Germany), and the thermal conductivity was calculated from the above-evaluated results. The density required for calculating thermal conductivity was measured using Archimedes’ law.

A universal testing machine (UTM; Instron 4464, US) was used to evaluate mechanical analysis with an applied load of 2 kN. Tensile stress and strain of the sample were tested at a constant strain rate of 10 mm/min until complete failure. Dynamic mechanical analysis (DMA; Q800, TA Instrument, US) was used in tension mode. The specimens were tested at 0.01% strain and 1 Hz frequency. All specimens were heated from −100 to 100 °C at a rate of 2 °C/min.

For the electrical conductivity measurements, a 4-probe method and the 4-point probe were stuck to the surface of each sample with planar dimensions of about 30 × 30 mm to ensure good contact of the sample surface with the electrodes, and sheet resistance/resistivity and electrical conductivity were measured using a resistivity meter (Loresta-GX MCP-T700, Mitsubishi Chemical Analytech, Japan).

### 2.5 Joule Heating

For the Joule heating, each sample was cut with a size of 25 × 75 mm^2^ and then pasted with the conductive copper tapes onto both side ends to connect with a DC power supply (TDP-3010B, Toyotech, Japan), followed by applying a fixed voltage of 10 V and a current of about 0.05–14.6 A. The temperature changes were measured by acquiring infrared thermal images with an IR camera (E5, FLIR, US). For heating rates, the time-dependent temperature of each sample was measured at approximately 25 °C and under 30% relative humidity.

## 3 Results and Discussion

### 3.1 Homogeneous Dispersion of CNT in BIIR

To acquire a homogeneous CNT/BIIR solution, IPA and MEP were respectively used as a solvent and additive to disperse pristine CNTs completely, as reported previously.^27^ When CNTs are mixed with an excess amount of IPA, which has an amphiphilic structure and is mildly sonicated, the aggregated CNT bundles are dispersed stably in IPA. MEP, as a nonvolatile polymeric organosilicon material, can form thermodynamically stable complexes of CNT/IPA/MEP in the IPA solution.^28^ Then, hexane was used to dissolve the BIIR matrix. Although IPA and hexane are miscible, BIIR is insoluble in IPA. Therefore, the well-dispersed hexane solution of BIIR was slowly dropped into the nanohybrid complex of CNT/MEP in IPA. Then, an additional volume of hexane was added to achieve a volume ratio of 2:1 of hexane to IPA. The addition of excess hexane allows the complete dissolution of BIIR into the CNT mixture solution. The resulting CNT/BIIR solution, with different percentages of CNTs from 5 to 20 wt %, exhibited uniform dispersion with no sedimentation observed even after one month. This uniform dispersion of solid fillers ensures the qualified electrical-thermal properties of the CNT/BIIR composite materials, as well-dispersed conductive fillers prevent local resistance spikes. These CNT/BIIR composite materials from well-dispersed CNTs and BIIR solution provided excellent physicochemical properties, including high electrical and thermal conductivities.

SEM images of the CNT/BIIR composite in Figure 2 indicate that CNT nanofillers are homogeneously embedded in the BIIR matrix. It shows that more CNTs protruded as the CNT content increased from 5 to 20 wt %. In particular, when the CNT content increased from CNT0.25 to CNT0.50, the interconnected points of CNTs increased significantly. As the CNT content increases, the porosity of the CNT/BIIR composites also increases, as observed in the black area indicated by the white arrows in Figure 3. In particular, the pores in CNT1.00 are densely concentrated in a specific area because the CNTs restrict the movement of BIIR during the hot pressing process above the melting point (*T*_m_).

**Figure 2:**
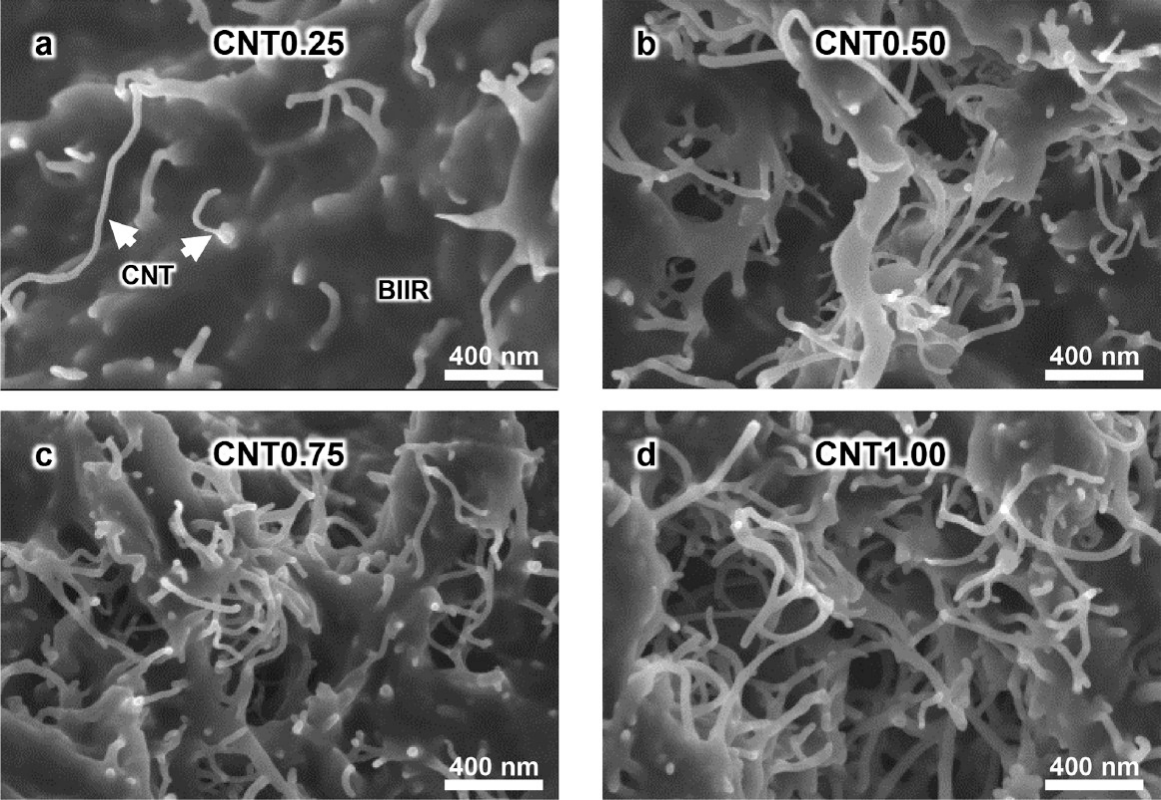
CNT structures in the CNT/BIIR composites. FE-SEM images of (a) CNT0.25, (b) CNT0.50, (c) CNT0.75, and (d) CNT1.00. Images were acquired under 50,000× magnification.

**Figure 3:**
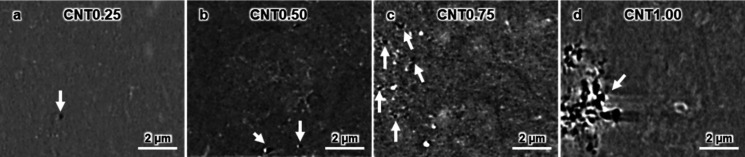
Cross-sectional XRM images of CNT/BIIR composites with different CNT concentrations. (a) CNT0.25, (b) CNT0.50, (c) CNT0.75, and (d) CNT1.00.

### 3.2 Thermal Analysis of CNT/BIIR Composite Film

The melting (*T*_m_) and degradation temperatures (*T*_d_) of the composite material were obtained from the DSC and TGA results, as shown in Figure 4 and summarized in Table 2. The pristine BIIR (CNT0) melts and completely thermally degrades at 117.65 and 400.34 °C, respectively. In Figure 4b, the residual weight is compared along with the initial CNT concentrations. The *T*_m_ of the composite material increased gradually from 122.58 to 126.92 °C with increasing CNT loading. It is known that the increase in glass transition temperature (*T*_g_) suggests that the interaction between the polymer chains of BIIR and the surface of CNT constrains the segmental motion of the BIIR chains.^29^ This is in the same tendency as the behavior of decomposition temperature. It indicated that the CNT fillers impart thermal stability to the composite material based on the strong interfacial adhesion between CNTs and the BIIR matrix. It is also notable that the activation energy required for thermal degradation of the composite increased with increasing content of CNT fillers, according to the DTG results in Figure 4c.

**Table 2:** Melting Point and Degradation Temperature of Composite Film

**sample name**	**melting point (*T***_**m**_, **°C)**	**degradation temperature (*T***_**d**_, **°C)**
**CNT0**	117.65	400.34
**CNT0.25**	122.58	404.85
**CNT0.50**	125.03	408.66
**CNT0.75**	125.70	409.10
**CNT1.00**	126.92	410.95

**Figure 4:**
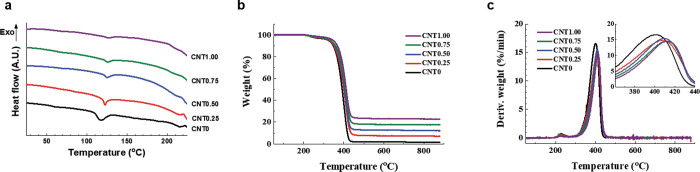
Changes in thermal stability with the CNT concentration. (A) DSC, (B) TGA, and (C) DTG.

As shown in Figure 5, both the thermal diffusivity and thermal conductivity increased linearly from 0.058 to 0.213 mm^2^/s and 0.041 to 0.217 W/m·K with increasing concentration of CNT from 0 to 20 wt %, respectively. This increase is attributed to the formation of a percolation network by increasing the number of CNT interconnection points, which makes the current path. This effect surpassed the scattering of phonons at the BIIR–CNT interfaces, which would otherwise interrupt heat transportation.^25^ Thermal diffusivity is also linearly proportional to the CNT concentration.

**Figure 5:**
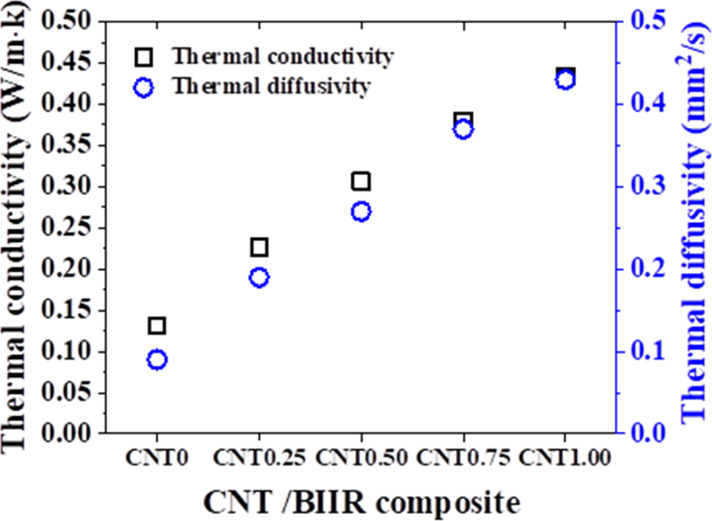
Thermal conductivity and thermal diffusivity of the CNT/BIIR film.

### 3.3 Mechanical Analysis of CNT/BIIR Composite Film

The mechanical property of the as-prepared composite films (hot pressed) was evaluated by a tensile test. In Figure 6a,b, it is clearly shown that the tensile strength increased linearly, but the elongation to break decreased rapidly with increasing content of CNT owing to the high stiffness of CNT. The reinforcement effect of CNTs in a composite material is well-known, derived from their superior property.^30^ Additionally, uniformly dispersed CNTs can form a network structure when they are overlapped through strong π-π and van der Waals interactions, leading to an enhanced stress transfer efficiency. Therefore, the tensile strength of BIIR was improved to 5.3 times with the loading of 20 wt % CNT (CNT1.00), but the increased porosity in CNT1.00 led to significantly reduced elongation at break (Figure 6a,b). Additionally, the CNT1.00 exhibited the disappearance of necking during the tensile test and linearly increased Young’s modulus up to 60 times (Figure 6a,b). This result is attributed not only to the high stiffness of CNT but also to the restriction of segmental movement in BIIR.^31^

**Figure 6:**
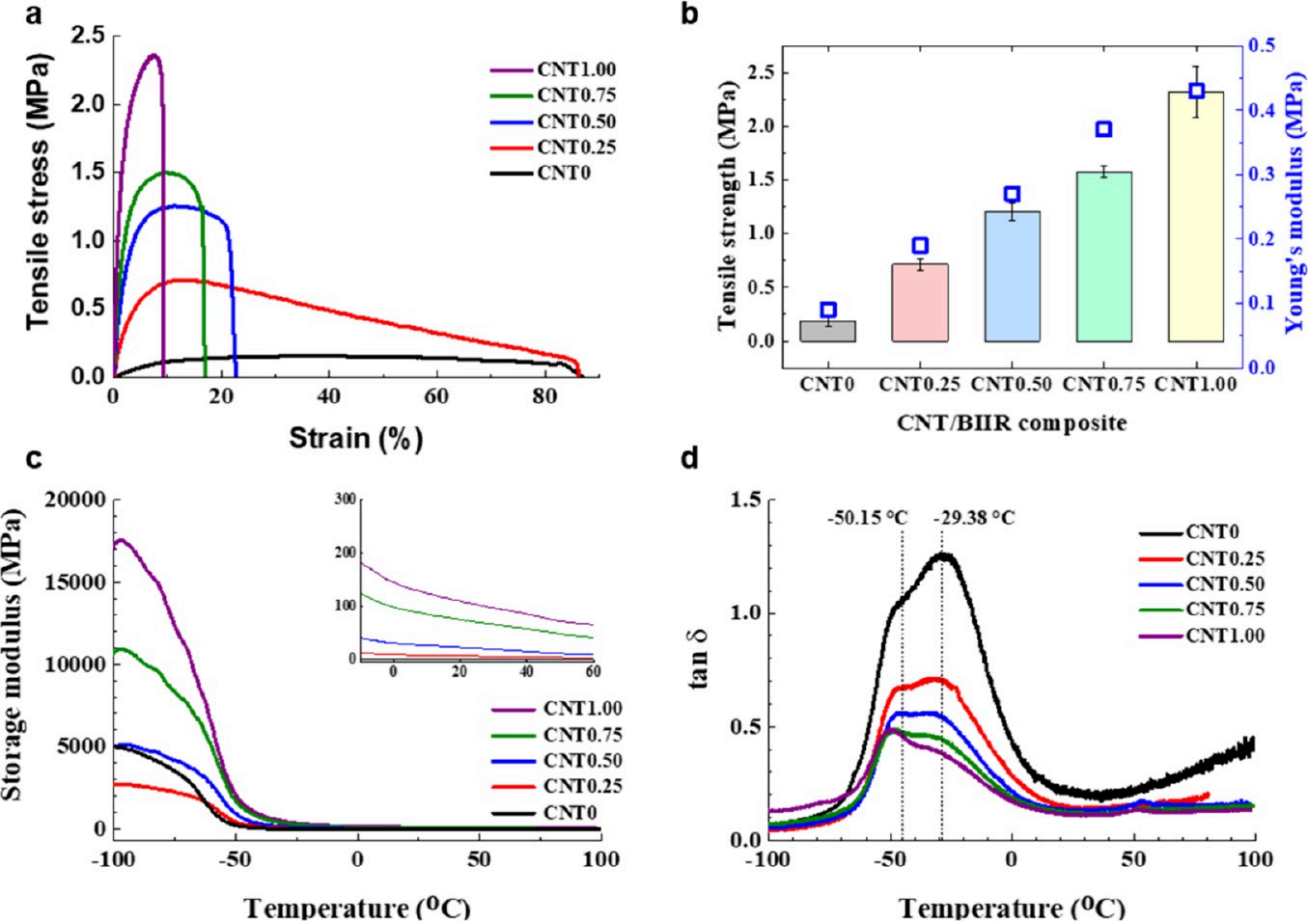
Tensile test: (a) stress–strain curve and (b) Young’s modulus. DMA: (c) storage modulus and (d) tan δ.

The effect of CNTs on the dynamic mechanical property of CNT/BIIR composites was characterized by DMA. The storage modulus and the loss factor (tan δ) of the neat BIIR and CNT/BIIR nanocomposites were investigated as functions of the temperature. As shown in Figure 6c, the storage modulus of the CNT/BIIR nanocomposites exceeds that of neat BIIR under all temperature ranges. The storage modulus refers to the recoverable strain energy in a deformed material. At low temperatures, it is clear that the stiffness of BIIR increased with increasing CNT addition, and the storage modulus decreased sharply with increasing temperature. The excellent mechanical properties and high specific surface areas of CNTs significantly improved the stiffness of the BIIR, which resulted in an increase in the storage modulus of the CNT/BIIR composite. The inset graph in Figure 6c, showing the rubbery plateau region, shows that CNTs restrict the polymer chain movement in the amorphous region.^32^ Figure 6d shows the tan δ as a function of temperature for neat BIIR and CNT/BIIR composites. The tan δ value of rubber is related to the energy dissipation as heat under oscillation. The position of the tan δ peak in the measured temperature range can be used to identify the *T*_g_ values of the polymer materials. The primary glass–rubber transition, referred to as α-transition, is characterized by a rapid decrease in storage modulus and the maximum of tan δ near the inflection point of the storage modulus. The α-transition occurs in the amorphous regions with the initiation of cooperative micro-Brownian motion of the molecular chains. The characteristic of secondary transition, which is referred to as β-transition, is that the less prominent value of tan δ maxima generally appears below the α-transition temperature. The β-transition is caused by the mobility of side groups or smaller unit backbone chains, and the transition range is broad and may appear as the shoulder of the α-transition. As shown in Figure 6d, the β-transition peaks were observed from all samples between −60 and −40 °C, and the α-transition peaks are also located between −40 and −20 °C in all samples. As the CNT content increased in the CNT/BIIR composites, the intensity of the β-transition peak increased compared to that of the α-transition peak. This phenomenon is attributed to the restriction effect of major molecular chains in the amorphous region of BIIR by CNT, whereas the movement of side groups and smaller unit chains is mainly activated.

### 3.4 Joule Heating Properties of CNT/BIIR Composite Film

Figure 7 shows the electrical properties of the CNT/BIIR films prepared with different contents of CNTs. The sheet resistances decrease exponentially from 35.95 ± 5.63 to 3.33 ± 0.20 Ω/sq with an increasing content of CNTs in the CNT/BIIR composite films. The electrical properties of each sample are summarized in Table 3. The CNT1.00 shows 7-fold higher electrical conductivity (3.93 ± 0.23 S/cm) than CNT0.25 (0.56 ± 0.08 S/cm). These results indicate that the well-dispersed CNTs in the BIIR matrix provide high electrical properties for the electrically conductive elastomeric composite film, preserving the homogeneous connections between CNTs and the effective percolation network of CNTs. The electrically conductive elastomer CNT/BIIR composite film showed extremely low sheet resistance (∼3.33 Ω/sq), indicating a better electron transfer structure for higher electrical conductivity and resulting in a highly stable and regular heating phase. These interconnected conductive networks enable Joule heating through the conversion of electrical energy into thermal energy, which represents a typical energy dissipation process in electro-thermal systems.^33^

**Table 3:** Electrical Properties of Each CNT/BIIR Composite Film

**sample name**	**surface resistivity (Ω/sq)**	**electrical conductivity (S/cm)**
**CNT0.25**	35.95 ± 5.63	0.56 ± 0.08
**CNT0.50**	10.45 ± 1.35	1.83 ± 0.25
**CNT0.75**	5.44 ± 0.29	2.86 ± 0.16
**CNT1.00**	3.33 ± 0.20	3.93 ± 0.23

**Figure 7:**
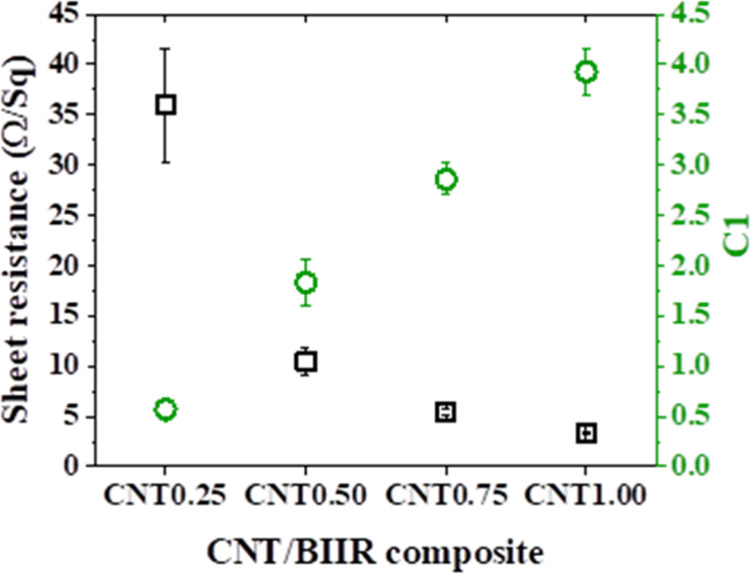
Electrical properties of the CNT/BIIR composite.

Figure 8a–d shows the heat distribution maps of each CNT/BIIR composite film when applying 10 V for Joule heating. The heat is uniformly distributed across the entire film surface, and the heat characteristics of each sample are summarized in Table 4. As shown in Figure 8e, the temperature increased rapidly during the initial stage (within 100 s) and gradually reached a plateau after approximately 150 s. The saturated temperature of the CNT0.25 film was 49 °C, whereas that of the CNT0.50 film significantly increased to approximately 119 °C. Notably, the CNT0.50 film exhibited a high heating rate of 102.2 ± 21.9 °C/min, calculated from the initial slope (0–30 s) of the temperature–time curve. This sharp increase can be attributed to the formation of an effective percolation network, where the density of conductive pathways is significantly enhanced, leading to improved electrical conductivity and Joule heating performance.^34^ These results indicate that the CNT/BIIR films have a high potential for self-healing applications accompanied by Joule heating. Figure 8f shows the temperature and power density obtained through Joule heating, and power density is calculated by Joule’s law (eq 1).^35^

$$P=V^{2}/R$$ 1

Where *P* is the power density (W) generated by Joule heating; *V* is voltage; and *R* is resistance. Since the resistance of the CNT/BIIR composite films decreased as the CNT contents increased, the CNT1.00 film exhibited the highest temperature at the same applied voltage.

**Table 4:** Summary of the Heating Characteristics of Each CNT/BIIR Composite Film

**sample name**	**CNT/BIIR (wt %)**	**applied power (W)** ^a^	**time to reach 100 °C (s)**	**heating rate (°C/min)** ^b^	**Max. temp. after 5 min (°C)** ^c^
**CNT0.25**	5	0.5 ± 0.1	N/A	41.4 ± 0.4	46.5 ± 1.5
**CNT0.50**	10	3.8 ± 0.3	101.7 ± 33.0	102.2 ± 21.9	115.0 ± 4.4
**CNT0.75**	15	8.5 ± 1.2	65.3 ± 22.6	85.8 ± 18.5	162.7 ± 32.0
**CNT1.00**	20	14.5 ± 0.1	32.0 ± 6.1	97.3 ± 3.9	189.7 ± 5.9

a Applied DC power was calculated from the fixed voltage and the current on each sample. The current was changed according to its resistance (*n* = 3).

b Heating rates were measured and calculated with the averages in box regions (*n* = 2).

c Maximum temperature was measured in the middle of the films (*n* = 3).

**Figure 8:**
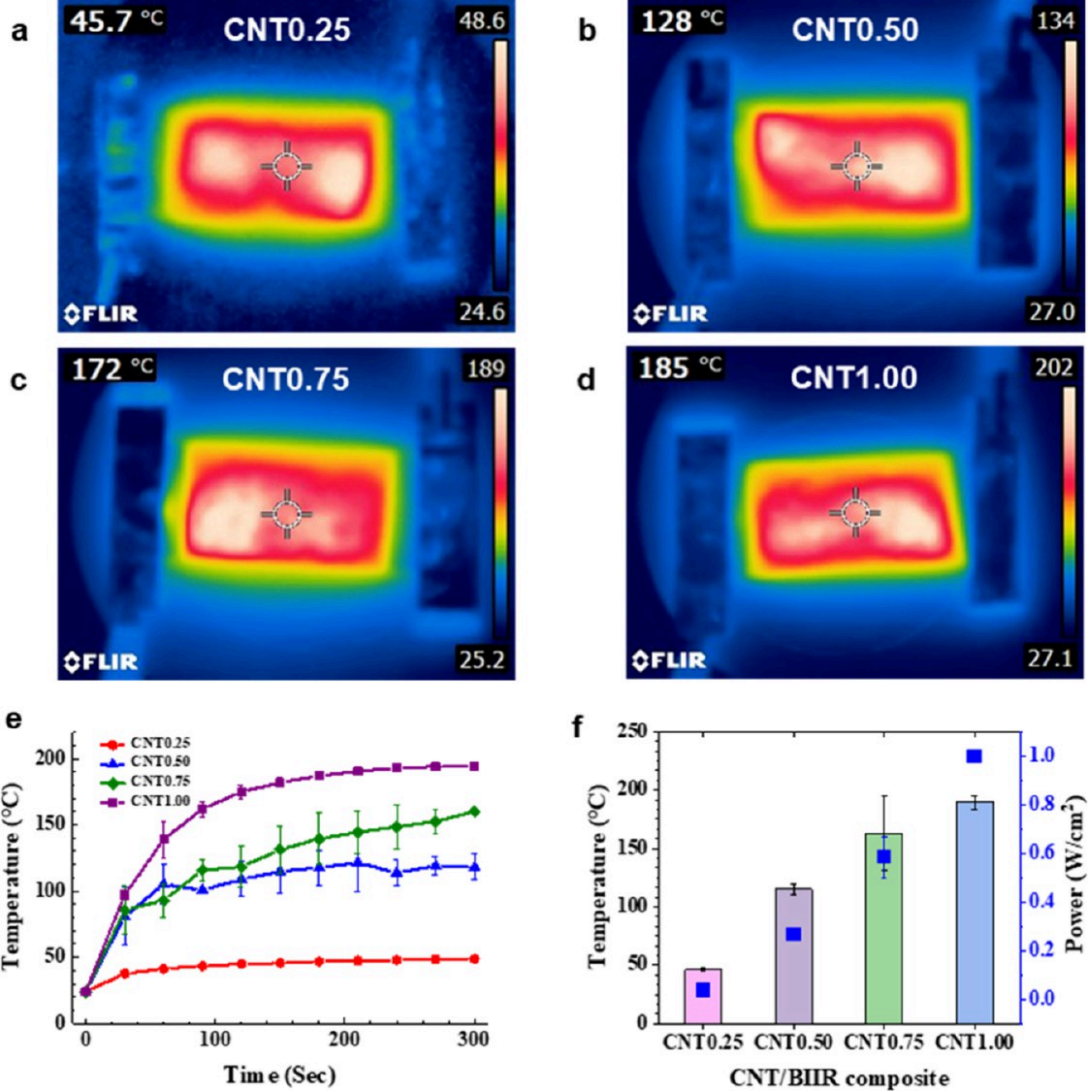
Heat distribution maps of (a) CNT0.25, (b) CNT0.50, (c) CNT0.75, and (d) CNT1.00 films. Electrical heating behavior of the CNT/BIIR composite films according to the CNT content in the BIIR matrix at 10 V. (e) Time-dependent temperature profiles and (f) temperature (bars) and power density (blue squares) after 5 min of Joule heating.

Alternatively, 15 mL of the CNT/BIIR solution was air-sprayed onto a polyimide film substrate for 1 min to form a film. Digital and IR images of the coated films are shown in Figure 9a,b. The sprayed film with S-CNT0.50 shows much higher film quality and uniform heat generation than that with S-CNT0.25, as shown by the IR images. Figure 9c shows the hand model and Joule heating result after air-spraying with 150 mL of S-CNT0.50. The spraying method is a promising way to form a uniform and thin film on curved surfaces, in comparison with drop casting and brush painting. It is well-known that the proper removal of solvent during the coating process is as important as the dispersion or antiprecipitation of the solute. In the spray process, the same volume of solvent is rapidly evaporated when the sprayed solution is atomized into a smaller size and higher surface area. Under an optimized condition, the excessive amount of solvent is evaporated from the droplet during delivery from the nozzle, and only the fine solutes are delivered to the substrate, forming a uniform film. In Figure 9c, 150 mL of S-CNT0.50 was air-sprayed onto the hand mannequin directly for 5 min. The nozzle-to-substrate distance was set to 5 cm. After drying the coating layer for 10 min, it was electrically connected by simply contacting the alligator clip and heated at 30 V and 0.24 A conditions. As seen in the images, the curved surface of the palm is coated with a black CNT/BIIR film and heated gradually by Joule heating (Figure 9c). This result shows that the CNT/BIIR solution is capable of coating directly on a desired object using a simple air-spray method with prompt adhesiveness and fair Joule heating properties.

**Figure 9:**
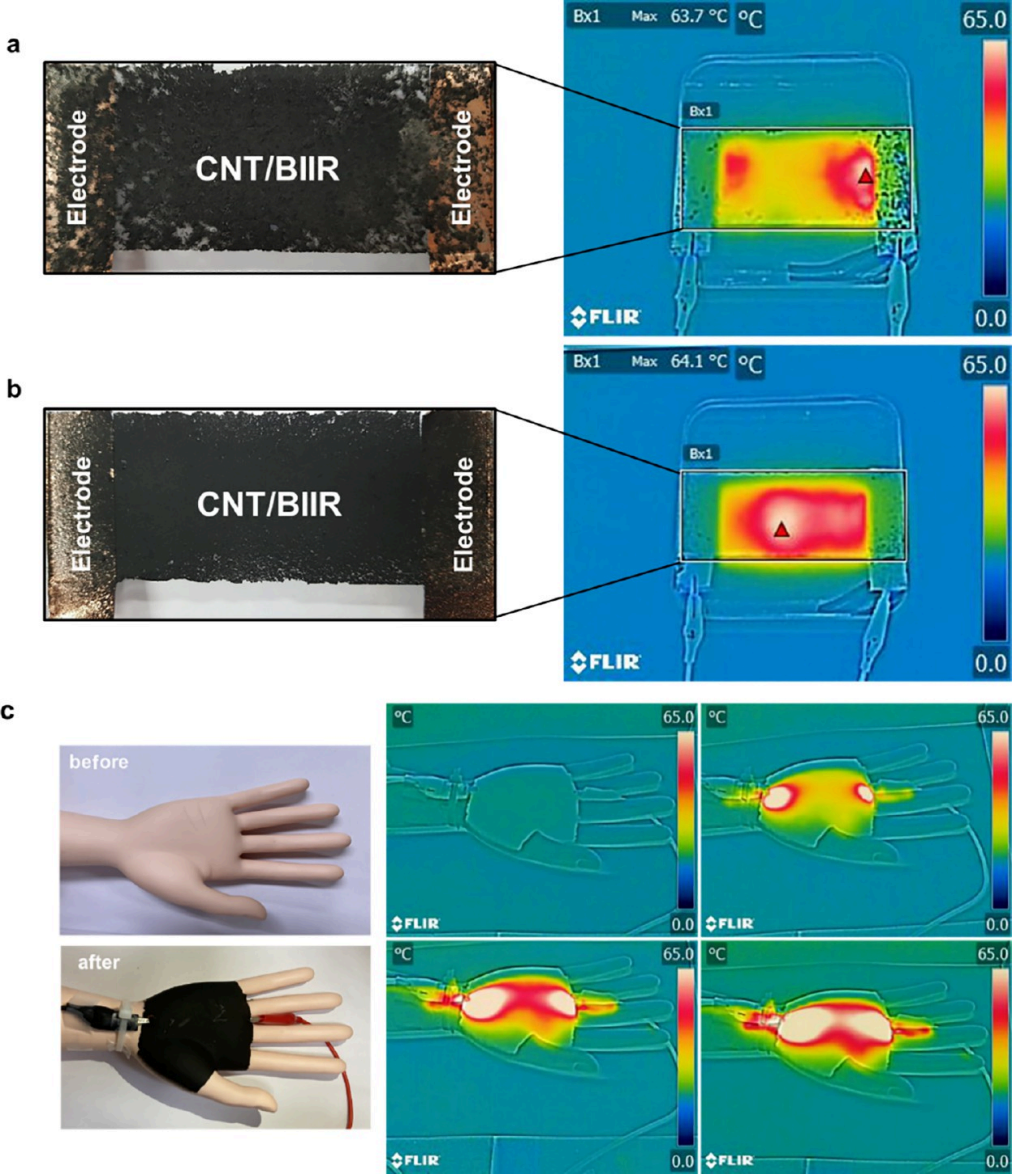
Air-sprayed film from a CNT/BIIR solution of (a) S-CNT0.25 and (b) S-CNT0.50. Voltage: 30 V, current: 0.08 A (S-CNT0.25), 0.09 A (S-CNT0.50). (c) Air-sprayed S-CNT0.50 on hand mannequin. Voltage: 30 V, current: 0.24 A.

### 3.5 Repair of CNT/BIIR Composite Films by Air-Spraying Coating

Figure 10 shows the air-sprayed self-healing results of the CNT0.50 composite film. The specimen size is 40 × 100 × 0.4 mm, and copper tape (10 mm wide) was attached to both ends of the specimen with silver paste (Figure 10a). 80 mL of S-CNT0.50 solution was air-sprayed half on each side of the composite at 25 °C for 5 min (Figure 10b). After air blast repair in Figure 10c, the 0.8 mm thick specimen shows that the cut marks are entirely covered and are not noticeable. In order to evaluate the repair behavior, a signal-based approach was employed, where variations in Joule heating were used to monitor the disruption and restoration of conductive pathways. The IR images showed that the composite film exhibited a uniform heat distribution through Joule heating before cutting (Figure 10d). With cutting, the maximum temperature increased at the sawtooth points, indicating a localized resistance increase due to the cut (Figure 10e). After self-healing with Joule heating, the composite film showed a homogeneous heat distribution again (Figure 10f). Figure 10g depicts the change in the maximum temperature over time when about 2.6 W was applied to the CNT/BIIR composite over 5 min. The result indicates that the heating rates of the control and healed samples are almost identical, suggesting that the spray coating covered the cracks well and stabilized them electrically and thermally. Figure 10h reveals that the electrical conductivity of the control sample is 0.6 S/cm, while the healed sample exhibits a conductivity of 2.2 S/cm, indicating a 3.7-fold increase compared to the control. This increase suggests that the conductive network was not only restored after healing but also further enhanced by the formation of additional CNT pathways. These pathways were likely formed through direct CNT bridging over the electrodes, along with the introduction of 0.062 g of CNT during the spray coating process. However, as shown in Figure 10i, the tensile strength and Young’s modulus of the control specimen are reduced by about half due to the severe cracks, but there is no significant enhancement after spray coating. The cut edges likely did not physically contact or rebond during the spraying process because the repaired CNT/BIIR composite was not heated to the melting point, which could have mended the edges of the crack and facilitated the healing mechanism. Therefore, heating above the melting point followed by cooling is expected to enhance the healing process further and increase the tensile strength. These results demonstrate that the restoration of electrical and thermal pathways enables the quantitative evaluation of repair efficacy. This signal-based response suggests that the proposed approach can be extended to structural-scale systems requiring continuous damage monitoring and repair.^36^

**Figure 10:**
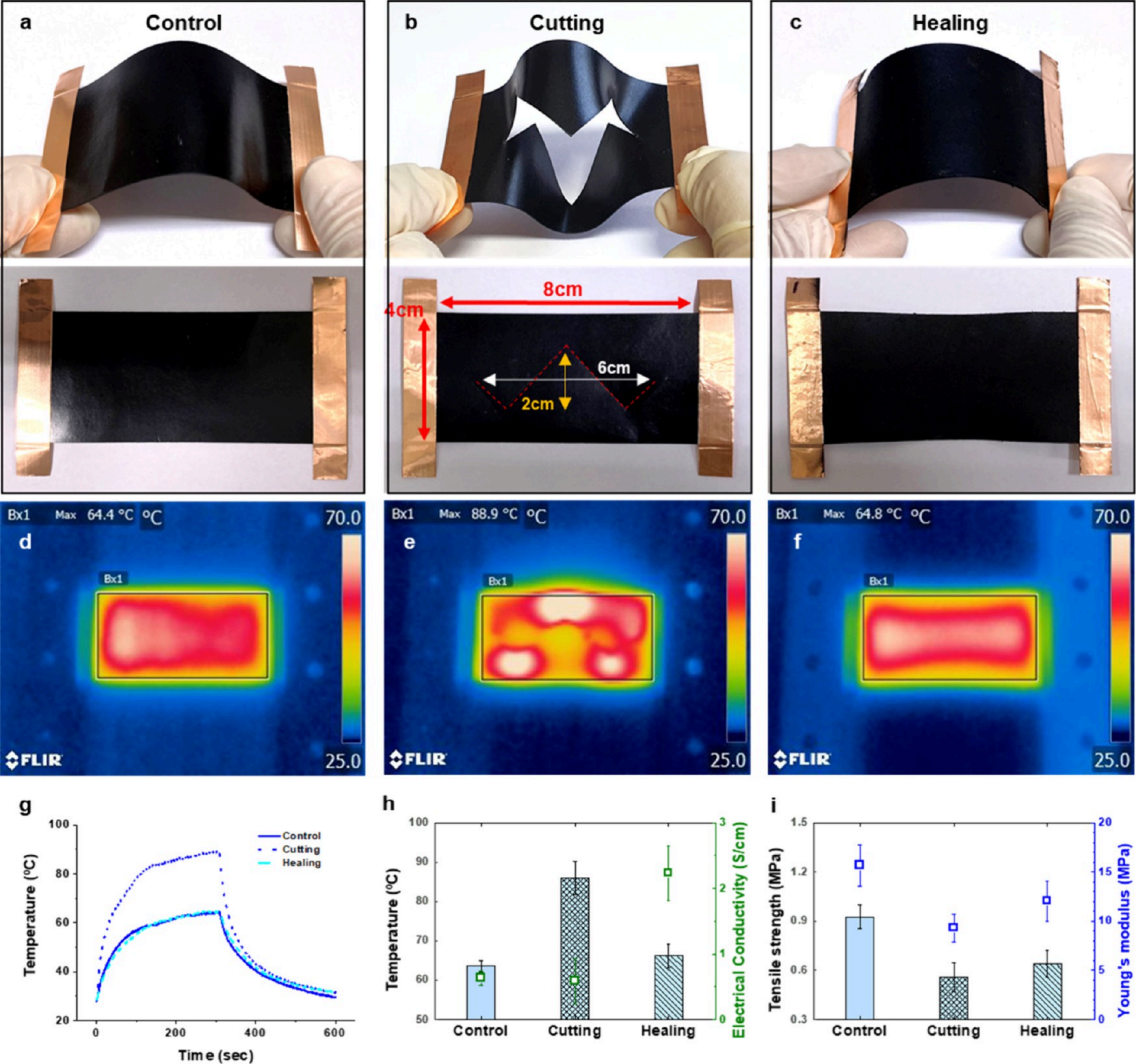
(a–c) Optical and (d–f) infrared camera images for the self-healing test of CNT0.50 film sprayed with S-CNT0.50. Voltage: 14 V, current: 0.19 A (control), Voltage: 16 V, current: 0.16 A (cutting), Voltage: 6 V, current: 0.43 A (healing). (g) Time-dependent temperature profiles, (h) maximum temperature after 5 min of power supply (bar), electrical conductivity (green square), and (i) tensile strength (bar) and Young’s modulus (blue square) for the self-healing test of 10% CNT/BIIR film.

## 4 Conclusions

In this study, the CNT/BIIR composites made by hot pressing or solution spraying methods were fully characterized to reveal their thermal, electrical, and mechanical properties. Despite the high CNT concentration up to 20 wt % in the BIIR matrix, CNTs were uniformly dispersed in the BIIR matrix by using IPA and MEP as a solvent and dispersant. The increase in the CNT concentration resulted in more cross-points acting as electronic paths and force transfer points, which enhanced the thermal conductivity, electrical conductivity, and tensile strength. The heat generation using the Joule heating effect and repair ability was also confirmed. The heating rate and maximum heating temperature of the CNT/BIIR composite were investigated with varying electrical properties at different CNT concentrations. Furthermore, the air-spraying method allowed for coating curved surfaces with a CNT/BIIR composite film while maintaining heat generation characteristics. The repair by the spraying method does not achieve complete physical healing; however, the current path is well recovered, resulting in uniform overall Joule heating. The demonstrated spraying repair method is a simple and cost-effective solution, and it holds promise for enhanced healing through Joule heating, leading to improved material performance and durability.
